# Efficacy of ultrasound-guided erector spinae plane block on analgesia and quality of recovery after minimally invasive direct coronary artery bypass surgery: protocol for a randomized controlled trial

**DOI:** 10.1186/s13063-024-07925-0

**Published:** 2024-01-19

**Authors:** Ling Xin, Lu Wang, Yi Feng

**Affiliations:** Department of Anesthesiology, Peking University People’s Hospital, No.11 Xizhimen South Street, Xicheng District, Beijing, China

**Keywords:** Erector spinae plane block, Postoperative pain, Nerve block, Minimally invasive, Randomized controlled trial

## Abstract

**Background:**

Minimally invasive direct coronary artery bypass (MIDCAB) surgery offers an effective option for coronary artery disease (CAD) patients with the avoidance of median sternotomy and fast postoperative recovery. However, MIDCAB is still associated with significant postoperative pain which may lead to delayed recovery. The erector spinae plane block (ESPB) is a superficial fascial plane block. There have not been randomized controlled trials evaluating the effects of ESPB on analgesia and patient recovery following MIDCAB surgery. We therefore designed a double-blind prospective randomized placebo-controlled trial, aiming to prove the hypothesis that ESPB reduces postoperative pain scores in patients undergoing MIDCAB surgery.

**Methods:**

The study protocol has been reviewed and approved by the Ethical Review Committee of Peking University People’s Hospital. Sixty adult patients of either sex scheduled for MIDCAB surgery under general anesthesia (GA) will be included. Patients will be randomly allocated to receive either a preoperative single-shot ESPB with 30 mL of ropivacaine 0.5% (ESPB group) or normal saline 0.9% (control group). The primary outcomes are the difference between the two groups in numeric rating scale (NRS) scores at rest at different time points (6, 12, 18, 24, 48 h) after surgery. The secondary outcomes include NRS scores on deep inspiration within 48 h, postoperative hydromorphone consumption, and quality of patient recovery at 24 h and 48 h, using the Quality of Recovery-15 (QoR-15) scale. The other outcomes include intraoperative fentanyl requirements, the need for additional postoperative rescue analgesics, time to tracheal extubation and chest tube removal after surgery, incidence of postoperative nausea and vomiting (PONV) and postoperative cognitive dysfunction (POCD), intensive care unit (ICU) length of stay (LOS), hospital discharge time, and 30-day mortality. Adverse events will be also evaluated.

**Discussion:**

This is a novel randomized controlled study evaluating a preoperative ultrasound-guided single-shot unilateral ESPB on analgesia and quality of patient recovery in MIDCAB surgery. The results of this study will characterize the degree of acute postoperative pain and clinical outcomes following MIDCAB. Our study may help optimizing analgesia regimen selection and improving patient comfort in this specific population.

**Trial registration:**

The study was prospectively registered with the Chinese Clinical Trial Registry (trial identifier: ChiCTR2100052810). Date of registration: November 5, 2021.

## Introduction

### Background and rationale {6a}

Over the past 10 years, minimally invasive direct coronary artery bypass (MIDCAB) surgery has been gaining popularity with smaller skin incisions and surgical trauma than conventional sternotomy coronary artery bypass grafting (CABG) [1]. The MIDCAB provides an effective alternative to coronary artery disease (CAD) patients requiring single or multi-vessel CABG through left anterior thoracotomy between the ribs [2]. It does not increase the risk of perioperative adverse cardiovascular and cerebrovascular events or 30-day mortality with reduced postoperative pain, faster recovery, and improved quality of life [3, 4]. However, MIDCAB surgery still causes moderate to severe postoperative pain that is related to thoracotomy, intercostal retraction, and chest drainage tubes.

Poorly controlled acute pain may lead to increased cardiopulmonary complications, delayed recovery, and prolonged intensive care unit (ICU) and hospital length of stay (LOS) as well as higher risk of developing chronic pain [5]. Therefore, the optimum method for postoperative analgesia is of critical importance in patients undergoing MIDCAB.

Postoperative pain management in cardiac surgery has traditionally been based on high-dose systemic opioids, which are effective but also responsible for kinds of feared effects including respiratory depression, nausea and vomiting, urinary retention, or constipation [6]. Regional anesthesia techniques are key components of multimodal analgesia in a variety of surgical settings. Thoracic epidural analgesia (TEA) and paravertebral block (PVB) have been applied as standard regional analgesia for thoracotomy. However, the use of these blocks in cardiac surgery has been controversial with consideration of the possible hemodynamic instability due to extensive sympathetic block. Moreover, intraoperative heparinization and perioperative antiplatelet therapy may result in an increased risk of serious block-related complications such as epidural or paravertebral hematoma. As a potential alternative, ultrasound-guided interfascial plane blocks have become increasingly popular as they are considered to be safer for cardiac surgeries with easier techniques to perform than TEA and PVB [7].

Erector spinae plane block (ESPB) is a superficial musculofascial plane block that injects local anesthetics into the space between the erector spinae muscles (ESM) and the transverse processes (TP) [8]. The efficacy of ESPB has been proven in thoracic and breast surgeries [9, 10]. In cardiac surgeries, 1 before-and-after study and 1 retrospective case–control study were conducted in adult cardiac patients using ESPB as a technique to manage acute postoperative pain [11, 12]. Only three randomized controlled trials of bilateral ESPB on postoperative analgesia for open cardiac surgery have been reported [13–15]. Besides, ESPB has also been shown to provide effective analgesia for mini-thoracotomy valve replacement surgery and left ventricular assist device implantation in observational studies and case series [16–18].

However, to our knowledge, no randomized controlled trials have evaluated this block in terms of analgesic effects or short-term and long-term outcomes among patients undergoing MIDCAB surgery. Here, we describe a double-blind prospective randomized placebo-controlled study, aiming to evaluate the effects of a preoperative unilateral ultrasound-guided single-shot ESPB on analgesia and quality of recovery among MIDCAB patients.

### Objectives {7}

The primary goal of this study is to evaluate the efficacy of preoperative single-shot ESPB on postoperative analgesia within 48 h compared with placebo after MIDCAB surgery.

Our secondary goals are to investigate whether the use of ESPB:

1. Promotes quality of patient recovery;
2. Reduces intraoperative opioids requirements and postoperative analgesic needs;
3. Facilitates early extubation and chest tube removal;
4. Shortens ICU stay and hospital discharge time; and
5. Decreases 30-day mortality.

### Trial design {8}

We are conducting a single-center, parallel-group, randomized controlled exploratory trial, with a 1:1 allocation of participants randomized to the intervention and control groups.

## Methods: participants, interventions, and outcomes

### Study setting {9}

The study site is Peking University People’s Hospital, a university-affiliated teaching hospital in Beijing, China.

### Eligibility criteria {10}

The inclusion criteria are as follows: adult patients from 18 to 75 years of either sex will be scheduled for elective MIDCAB surgery under general anesthesia (GA). The exclusion criteria are as follows: New York Heart Association (NYHA) grading IV, preoperative ejection fraction (EF) < 40%, body mass index (BMI) < 18 or > 35, redo or emergency surgery, coagulopathy, localized infection at the block site, allergy or intolerance to local anesthetics, hepatic or renal insufficiency, diagnosed mental disorder, history of chronic pain conditions requiring opioid usage, and patient refusal.

### Who will take informed consent? {26a}

The principal investigator will assess eligible patients to participate in this study based on the inclusion and exclusion criteria. The research team members will introduce the study protocol to the participants. If the patient is willing to participate in this study, a written informed consent will be given for the patient’s signature.

### Additional consent provisions for collection and use of participant data and biological specimens {26b}

Not applicable. No biological specimens of the participant will be collected during the trial.

## Interventions

### Explanation for the choice of comparators {6b}

The novel ESPB has to be evaluated for analgesic efficacy in MIDCAB surgery. The choice of interventions will help us to achieve valuable information for a better understanding of the impact of this regional technique on pain control. The intravenous (IV) patient-controlled analgesia (PCA) and rescue analgesics will serve as standard postoperative analgesia and prevent therapeutic gaps in case of ineffective block.

## Intervention description {11a}

### Standard perioperative management

All patients will receive GA for MIDCAB surgery. A radial arterial catheter will be inserted for continuous invasive blood pressure (IBP) monitoring. Induction of GA will be a combination of IV midazolam (0.03–0.05 mg/kg), etomidate (0.15–0.2 mg/kg), fentanyl (3–5 μg/kg), and cis-atracurium (0.15–0.2 mg/kg), and then an appropriate sized left-sided double-lumen tube will be inserted. Mechanical ventilation will be performed in volume control mode. The respiratory frequency will be adjusted to achieve end tidal carbon dioxide (ETCO_2_) between 30 and 35 mmHg. A 7-Fr triple-lumen central venous catheter will be placed after induction under ultrasound guidance to monitor central venous pressure (CVP). Continuous infusion of propofol (3–5 mg/kg), cis-atracurium (0.1–0.2 mg/kg/h), and dexmedetomidine (0.5–0.8 μg/kg/h) will be used for anesthesia maintenance with additional fentanyl boluses according to bispectral index (BIS) value and hemodynamic status.

During the surgical procedure, the BIS value will be kept between 40 and 60 and IBP within ± 20% of the baseline value. In case of hypertension (defined as IBP > 20% of baseline value), the attending anesthesiologist will check the anesthesia depth and volume status of the patient, then increase propofol infusion rate by 1 mg/kg and/or administer fentanyl bolus by 1 μg/kg or nicardipine 0.1–0.3 mg. Hypotension (IBP < 20% of baseline value) will be corrected by decreasing propofol by 1 mg/kg and/or fluid volume loading by 3 mL/kg. Continuous infusion of phenylephrine (0.3 μg/kg/min) or dopamine (3 μg/kg/min) will be initiated if normal IBP is still not achieved. Tachycardia (heart rate > 80 bpm) will be treated with esmolol bolus by 0.5 mg/kg, while bradycardia (heart rate < 45 bpm) will be treated with anisodamine by 1–3 mg, respectively. Intraoperative patient nasopharyngeal and urinary bladder temperature as well as urine output will also be monitored.

PCA with IV hydromorphone will be prescribed for all patients with no basal infusion and an intermittent bolus dose of 0.2 mg, with a lock-out period of 10 min. IV tropisetron 5 mg will be used at the end of surgery for postoperative nausea and vomiting (PONV) prophylaxis.

All IV anesthetic infusions including sedatives and analgesics will be stopped upon completion of surgery; afterwards, the patients will be transferred to the cardiac ICU whereby postoperative hemodynamic and respiratory parameters are evaluated. No sedatives or analgesic infusions will be administered in the cardiac ICU until the patient is extubated. Patients will be extubated when the following weaning criteria are met: patients are conscious with spontaneous breath in pressure support (PS) mode of 6–8 cmH_2_O and FiO_2_ < 0.4, respiratory rate between 12 and 20 breaths/min, PaO_2_ ≥ 60 mmHg, PaCO_2_ ≤ 45 mmHg, no need for inotrope use to maintain mean arterial pressure (MAP) ≥ 60 mmHg, urine output > 0.5 mL/kg/h, no signs of active bleeding, and the amount of drainage from chest tube reaching to 50 mL/h.

If the patient required, extra bolus of hydromorphone 0.4 mg (up to 1.2 mg per hour) will be delivered via PCA device. IV fentanyl 50 μg will be used for rescue analgesia in case the numeric rating scale (NRS) score at rest is still equal or greater than 4 or the patient demands additional analgesia. No any other specific protocol will be used by the cardiac ICU staff to manage analgesia after surgery. Forty-eight hours postoperatively, PCA will be discontinued and data will be transmitted from the pump to the institutional analgesia database. After being transferred to the ward, further analgesic treatment with oxycodone and acetaminophen will be prescribed for the patient if necessary.

### ESPB

All the ultrasound-guided ESPB will be performed by the same investigator (LX) who is experienced in ESPB before GA induction with patients under sedation by IV midazolam 1–2 mg. Patients allocated to the ESPB group will receive a single-shot of 0.5% ropivacaine 30 mL at the T5 level under local anesthesia, whereas the control group will receive 0.9% normal saline 30 mL at the same block site.

The patients will be placed in the right lateral position. We will use chlorhexidine gluconate to disinfect the skin before performing the block procedure to prevent pathogen contamination. After disinfection of the skin, a low-frequency linear transducer will be initiated in a paramedian sagittal plane at the T5 level, approximately 2–3 cm lateral to the posterior midline. Then, the posterior lateral apex of the TP will be identified as a landmark for the block. Whereafter, the probe will be adjusted to visualize the following three muscles: trapezius muscle (TM), rhomboid muscle (RM), and ESM. Then, a 100-mm echogenic block needle will be inserted under local infiltration anesthesia using the in-plane technique from the cephalad. The needle tip will be advanced to reach the interfascial space between the TP and ESM. The correct position of the needle tip will be confirmed with 1–2 mL normal saline; then, 30 mL of the study medication will be injected.

### Criteria for discontinuing or modifying allocated interventions {11b}

Discontinuation criteria are as follows: surgery is converted to median sternotomy, patients who undergo redo surgery before hospital discharge, patients who are unconscious or unable to cooperate at the time points of postoperative follow-up, and patients who decide to leave the study. The patient has the right to withdraw the informed consent and cease participation in the study at any time. Participants who leave early will be provided with local standard of care for MIDCAB surgery.

### Strategies to improve adherence to interventions {11c}

Prior to the start of the study, cardiac anesthesiologists in charge of the surgery will receive information about the study protocol and will be trained in the handling of perioperative management, which will improve protocol adherence. Apart from the intervention, the participants will receive standard care during hospital admission.

The investigators will make best effort to do a close follow-up and good communication with the participants throughout the study period. The participants will be provided with detailed study timeline before data collection and forenotice of the upcoming follow-up visit. Any question or concern related to the study and clinical treatment from the participants will be responded patiently in detail.

### Relevant concomitant care permitted or prohibited during the trial {11d}

Any interventions or concomitant care that may influence the study outcomes are prohibited, such as other type of regional blocks.

### Provisions for post-trial care {30}

The single-shot ESPB is a relatively safe technique with no block-related complications that has been reported in meta-analyses [19, 20]. Thus, the participants are not expected to suffer injury from participating in the trial. If any harm was occurred, the patient will be treated by appropriate departments in our hospital.

### Outcomes {12}

The primary outcome is pain intensity measured by the NRS score at rest at different time points (6, 12, 18, 24, 48 h) after MIDCAB. The NRS is a self-reported 11-point scale representing different degrees of pain. The number 0 represents painless, while 10 represents worst pain ever. The intensity of pain can be categorized into mild (1 to 3), moderate (4 to 6), and severe (7 to 10). The secondary outcomes are NRS scores on deep inspiration within 48 h after surgery, postoperative 48 h hydromorphone consumption, and quality of patient recovery at 24 h and 48 h, assessed by the Quality of Recovery-15 (QoR-15) scale. Other outcomes include intraoperative fentanyl requirements, additional use of postoperative rescue analgesics, time to tracheal extubation and chest tube removal after surgery, LOS in ICU, and hospital discharge time. Adverse events including PONV, postoperative cognitive dysfunction (POCD), atrial fibrillation, pleural effusion, pericarditis, and block-related complications, for instance, hematoma, pneumothorax, and infection, will be recorded. Thirty-day mortality will also be evaluated as a safety outcome.

### Participant timeline {13}

**Fig. 1:**
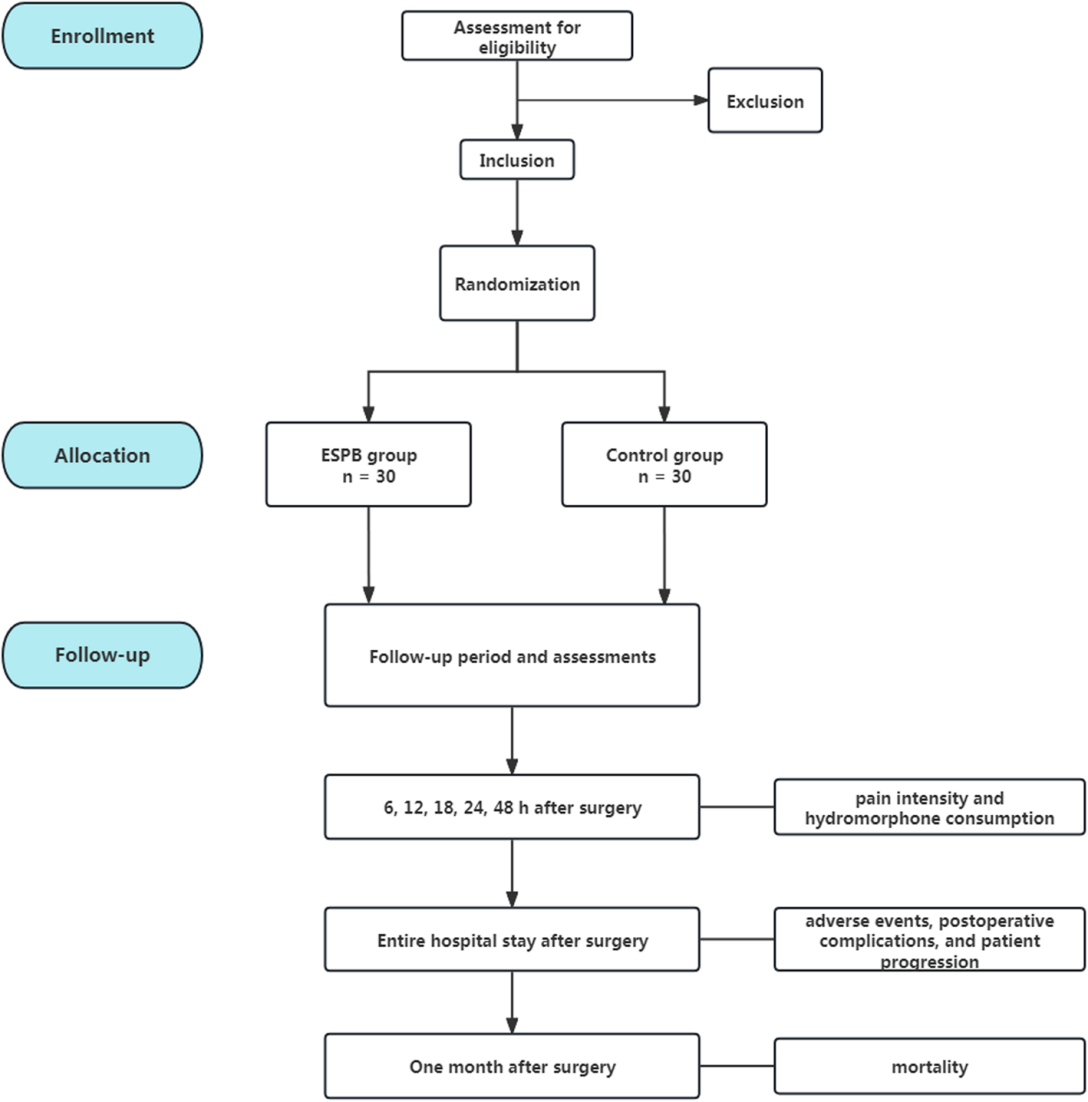
Flowchart of the trial. −t_1_, preoperative; t_1_, before induction of general anesthesia; t_2_, completion of surgery; t_3_, ICU admission; t_4_, 6 h after surgery; t_5_, 12 h after surgery; t_6_, 18 h after surgery; t_7_, 24 h after surgery; t_8_, 48 h after surgery days after surgery; t_9_, 30 days after surgery; ESPB, erector spinae plane block; NRS, numeric rating scale; QoR, quality of recovery

The time schedule of enrollment, interventions, and assessments for the participants of this study is presented in Table 1. The flow chart of the study is shown in Fig. 1.

**Table 1:** Checklist of enrolment, interventions, and assessments

	Study period										
	Enrolment	Allocation	Post-allocation								Close-out
Timepoint**	*-t*_*1*_	0	*t*_*1*_	*t*_*2*_	*t*_*3*_	*t*_*4*_	*t*_*5*_	*t*_*6*_	*t*_*7*_	*t*_*8*_	*t*_*9*_
**Enrolment:**											
**Eligibility screen**	X										
**Informed consent**	X										
**Allocation**		X									
**Interventions:**											
***ESPB***			X								
***Control***			X								
**Assessments:**											
***Baseline characteristics***	X	X									
***NRS for pain***						X	X	X	X	X	
***Hydromorphone consumption***									X	X	
***QoR-15***									X	X	
***Fentanyl requirements***				X							
***Rescue analgesics***											
***Adverse events***											
***Mortality***											X

### Sample size {14}

Sample size is determined on the basis of a preliminary study to observe the efficacy of ESPB on MIDCAB patients within 12 h after surgery in our hospital. We use the PASS 11.0 software to calculate the sample size. We power the study to identify a relevant clinical difference of 1.5 in NRS score with a standard deviation (SD) of 1.9 [21]. Thus, to compare two means using repeated measures analysis of variance (ANOVA) method with a power of 90% and a level of significance of 5% (two sided), a sample size of 46 patients is estimated in this study. Considering possible dropouts, a sample size of 60 patients will be required.

### Recruitment {15}

The principal investigator is responsible for patient recruitment. Every potential participant will be visited by investigator 1 day before operation in the ward and provided with details of the study, including study backgrounds, protocol, possible benefits and risks other medical choices, right to participate and withdraw, and use of individual information and data.

## Assignment of interventions: allocation

### Sequence generation {16a}

According to a computer-generated random number, patients will be allocated to either the ESPB group or the control group at a 1:1 ratio.

### Concealment mechanism {16b}

The group assignments will be sealed in sequentially numbered opaque envelopes and locked in a cabinet in the research office. Randomization information will not be unveiled until the envelope is opened.

### Implementation {16c}

A nurse anesthetist who is not involved in the treatment, evaluation, data collection, or statistical analysis will generate the allocation sequence. The nurse anesthetist will open the envelope 1 h prior to surgery and prepare the study medications accordingly. The study medications are 0.5% ropivacaine 30 mL for the ESPB group and 0.9% normal saline for the control group. Both syringes would be identical and will be labeled as “study medication” to ensure the blinding procedure.

## Assignment of interventions: blinding

### Who will be blinded {17a}

All participants will be blinded to group allocations. The anesthesiologists who will perform the block and those who will provide intraoperative care are blinded. A blinded investigator will collect the perioperative data. The cardiac surgeons and medical staff in cardiac ICU and the ward will be unaware of the group allocations.

### Procedure for unblinding if needed {17b}

In case of medical necessity or emergency related to the procedure, the principal investigator could decide to unblind and inform the patient and attending medical staff.

## Data collection and management

### Plans for assessment and collection of outcomes {18a}

Patient demographic data (age, weight, height, and ASA classification), medical history, results of lab tests, imaging exams, and echocardiogram will be achieved from the electronic medical record. Surgical and intraoperative events as well as other related perioperative anesthetic details will be documented. Postoperative pain severity will be evaluated after patient arrival in the ICU (0 h) at predetermined time points by follow-up investigators who are blinded to the study group allocations using the NRS score. Analgesic consumption and the QoR-15 scores will be collected on 24 h and 48 h postoperatively.

We will use the Chinese version of the QoR-15 questionnaire as a measurement of the patient’s recovery quality which contains the following domains: physical comfort and independence, pain, psychological, and emotional state [22]. The QoR-15 survey will be taken on the ward where the patient will be left alone to accomplish the questionnaire without disturbance.

POCD will be evaluated using MoCA on the day of enrollment and 3 days after surgery [23]. Adverse events, postoperative complications, and patient progression will be traced by visiting the patients daily or reviewing electronic medical records during their entire hospital stay. A telephone interview will be performed by LW at 1 month after surgery to assess 30-day mortality.

### Plans to promote participant retention and complete follow-up {18b}

The investigators will make best effort to do a close follow-up and good communication with the participants throughout the study period. The participants will be provided with detailed study timeline before data collection and forenotice of the upcoming follow-up visit. Any question or concern related to the study and clinical treatment from the participants will be responded patiently in detail.

### Data management {19}

All the participant’s relevant data will be initially documented on paper case report form (CRF). Paper CRFs will be subsequently converted into electronic files in excel format by one investigator (LW). All research data will be stored for 5 years after the study.

### Confidentiality {27}

All the electronic data and paper documents will be securely stored in the hospital’s information system and a locked file storage cabinet respectively for at least 5 years after the completion of the study. Only the investigators will be authorized to get access to the study dataset. The participants’ personal identifying information will be replaced by study identification codes for confidentiality.

### Plans for collection, laboratory evaluation, and storage of biological specimens for genetic or molecular analysis in this trial/future use {33}

Not applicable. There are no relevant plans since no biological specimens will be involved in the present trial.

## Statistical methods

### Statistical methods for primary and secondary outcomes {20a}

Statistical analysis will be done using SPSS Statistics (IBM: V. 24). Before analysis, the normality of the data distribution will be assessed using the Shapiro–Wilk test and Q–Q plots. For postoperative NRS scores, we will use repeated measurement with post hoc testing. For the other outcomes, postoperative hydromorphone consumption, QoR-15 scale, intraoperative fentanyl requirements, time to tracheal extubation and chest tube removal after surgery, LOS in ICU, and hospital discharge time will be analyzed with the independent samples *t* test or Mann–Whitney *U* test. The incidence of PONV, POCD, additional use of postoperative rescue analgesics, and 30-day mortality will be compared by the *χ*^2^ or Fisher’s exact test when necessary. All *P* values will be two sided, and statistical differences will be defined as less than 0.05. Missing data will be handled with multiple imputation methods.

### Interim analyses {21b}

Not applicable. No interim analysis is planned for the present trial.

### Methods for additional analyses (e.g., subgroup analyses) {20b}

Not applicable. There are no subgroup analyses planned.

### Methods in analysis to handle protocol non-adherence and any statistical methods to handle missing data {20c}

Data will be analyzed by both intention-to-treat and per-protocol principals. Missing data will be handled with multiple imputation methods.

### Plans to give access to the full protocol, participant level-data and statistical code {31c}

The corresponding author can be contacted for sharing participant-level dataset upon reasonable request.

## Oversight and monitoring

### Composition of the coordinating center and trial steering committee {5d}

The coordinating center is the Department of Anesthesiology of Peking University People’s Hospital, Beijing, China. The principal investigator (LX) will take full responsibility for the study. The trial steering committee consists of the principal investigator (LX) and co-investigators (LW and YF), who are responsible for recruiting patient recruitment, trial conduct, data collection, and entry.

### Composition of the data monitoring committee, its role and reporting structure {21a}

The data monitoring committee will be composed of the Ethical Review Committee of Peking University People’s Hospital, which is independent of the sponsor and competing interests. Data quality reporting will be conducted by the principal investigator.

### Adverse event reporting and harms {22}

In the present study, we define serious adverse events (SAEs) as one of the following criteria: patient death or any life-threatening conditions and persistent or significant disability. SAEs will be reported within 24 h to the quality and safety control committee and the ethics committee of our hospital. All adverse events related to this study will be recorded and reported to the ethics committee by the principal investigator within 1 week.

Systemic toxicity of local anesthetic, pneumothorax, hematoma, and injection site infection will be continuously inspected by physical examination, X-ray, and/or ultrasound in the operating room and ICU after the administration of study medication. Ultrasound guidance, negative aspiration for blood before local anesthetic injection, and low-speed administration of local anesthetic will be used to help prevent the occurrence of pneumothorax, hematoma, and systemic toxicity of local anesthetic.

### Frequency and plans for auditing trial conduct {23}

The research team will meet monthly to check the trial progress and discuss any issues that will be encountered during trial conduct. The trial conduct will be audited every 6 months by the Ethical Review Committee of Peking University People’s Hospital.

### Plans for communicating important protocol amendments to relevant parties (e.g., trial participants, ethical committees) {25}

Any amendment to the study protocol, informed consent, or other related documents will also be asked for approval by the ethics committee.

### Dissemination plans {31a}

The results of this study will be disseminated via academic conference presentations and publication in peer-reviewed journals. The findings and conclusions will be shared with all the participants upon completion of the study. The principal investigator of the study will be the lead author. The investigators that have taken part in the study for at least 3 months and contributed to the manuscript drafting will be listed in the authorship.

## Discussion

As more minimally invasive cardiac surgeries are being performed in recent years, the need for better postoperative pain management and faster recovery has become a pressing concern. Although the incision is smaller, MIDCAB surgery can still cause moderate to severe postoperative pain which could make the patient’s breathing shallow and irritating and result in increased risk of postoperative pulmonary complications (PPCs). PPCs may impede early recovery and are associated with prolonged hospital LOS and increased mortality following cardiac surgery [24, 25]. Therefore, optimizing analgesic regimen with multimodal approaches to achieve adequate pain relief following MIDCAB surgery is a crucial consideration.

In our hospital, ESPB has been successfully applied in thoracic and lumbar spinal surgeries for analgesia and its safety profile has been well documented in these studies [26, 27]. In addition, the volume and concentration of the study medication have been safely used in previous studies without any block-related complications reported [12, 28]. However, although the efficacy of bilateral ESPB has been demonstrated in adult cardiac surgery with midline thoracotomy, it is still unclear whether this technique is effective and beneficial for MIDCAB patients. Thus, we designed this randomized controlled study and expect to fill in a gap in the existing literature. The postoperative resting NRS score is the primary outcome and being measured at different time points during the first 48 h after surgery to better delineate pain intensity patterns. Additionally, perioperative opioid consumption and associated side effects as well as early postoperative recovery outcomes are also being measured. We anticipate that the findings of our study would help to clarify the impact of ESPB on analgesia and patient recovery following MIDCAB surgery and may also help to optimize analgesic regimen in this specific population.

We also foresee some limitations in this study. For instance, patients who have accidental prolonged progress after surgery may be unable to complete the NRS assessment at predetermined time points. As a result, a certain lost to follow-up bias cannot be ruled out. In addition, the dermatomal distribution of ESPB is not tested, and the optimal volume and concentration of ESPB is unevaluated, emphasizing the need for further investigations.

### Trial status

The protocol is version 3.0, dated October 7, 2021. The patient recruitment has been initiated on November 22, 2021, and is expected to last for 10 months.

## Abbreviations

MIDCAB: Minimally invasive direct coronary artery bypass
CAD: Coronary artery disease
ESPB: Erector spinae plane block
GA: General anesthesia
NRS: Numeric rating scale
QoR-15: Quality of Recovery-15
PONV: Postoperative nausea and vomiting
POCD: Postoperative cognitive dysfunction
ICU: Intensive care unit
LOS: Length of stay
CABG: Coronary artery bypass grafting
TEA: Thoracic epidural analgesia
PVB: Paravertebral block
ESM: Erector spinae muscles
TP: Transverse processes
NYHA: New York Heart Association
EF: Ejection fraction
BMI: Body mass index
IV: Intravenous
PCA: Patient-controlled analgesia
IBP: Invasive blood pressure
ETCO_2_: End tidal carbon dioxide
CVP: Central venous pressure
BIS: Bispectral index
PS: Pressure support
MAP: Mean arterial pressure
TM: Trapezius muscle
RM: Rhomboid muscle
SD: Standard deviation
CRF: Case report form
SAEs: Serious adverse events
PPCs: Postoperative pulmonary complications
